# Energy intake insufficiency due to underestimated energy requirement by common predictive formulas can be identified by urinary amino acid levels in advanced heart failure

**DOI:** 10.3389/fnut.2024.1504031

**Published:** 2025-01-23

**Authors:** Yoko Sakamoto, Tomohito Ohtani, Kei Nakamoto, Fusako Sera, Shungo Hikoso, Yasushi Sakata

**Affiliations:** ^1^Department of Cardiovascular Medicine, Osaka University Graduate School of Medicine, Suita, Japan; ^2^Cardiovascular Division, Osaka Keisatsu Hospital, Osaka, Japan; ^3^Department of Cardiovascular Medicine, Nara Medical University, Kashihara, Japan

**Keywords:** resting energy expenditure, urinary amino acids, histidine excretion, advanced heart failure, prediction model

## Abstract

**Background:**

Elevated resting energy expenditure (REE) promotes cachexia, worsening prognosis in patients with advanced heart failure (HF). However, adequate assessment of energy balance is challenging because of unvalidated common prediction methods and unestablished determinants of REE, resulting in a lack of biomarkers for predicting insufficient energy intake.

**Objective:**

This cross-sectional study aimed to evaluate REE in patients with advanced HF and explore biomarkers for insufficient energy intake.

**Methods:**

We measured REE by indirect calorimetry and calculated the total energy expenditure (TEE) of 72 hospitalized patients with advanced-stage HF. We compared these values with commonly-used formulas and analyzed the associations between REE per body weight (REEBW) and parameters related to hemodynamics and HF severity. In 17 of 72 patients, plasma amino acid (AA) and 24-h urinary AA concentrations were measured to analyze their correlations with energy balance, the ratio of caloric intake to REE.

**Results:**

Resting energy expenditure and TEE values were significantly higher than the predicted values. The mean REEBW was 25 kcal/kg/day, while that for the underweight (<18.5 kg/m^2^) was 28 kcal/kg/day. We found a significant negative correlation between REEBW and body mass index (BMI), but no significant correlation between REEBW and HF-related parameters. The difference between TEE and predicted TEE using the European Society for Clinical Nutrition and Metabolism formula was most significant in the underweight patients because of underestimation, whereas TEE and pTEE using our modified formula with coefficients by BMI categories did not differ. There was a significant correlation between energy balance and urinary histidine and its metabolite 3-methylhistidine excretion, but no significant correlation with serum albumin and other AA concentrations.

**Conclusion:**

Underweight patients with advanced HF require more energy per weight than the predicted value. Our proposed formula for pTEE in each BMI category may be useful in clinical practice to avoid underestimation of daily energy requirements. Inadequate energy intake, even with such an approach, may be identified by decreased urinary essential AA levels.

## Introduction

Cachexia is a critical issue in patients with advanced heart failure (HF) as it is associated with increased mortality (1). Multiple factors, including chronic inflammation, neuroendocrine activation, insulin resistance, malabsorption, anorexia, and elevated resting energy expenditure (REE), play a role in promoting cachexic changes in HF (2–4). REE can vary according to the severity and type of disease, including HF (5). A previous study reported that the average REEs in 59 patients with stage C HF was 22 kcal/kg in normal nutritional status and 24 kcal/kg in low nutritional status, respectively (6). However, available data on patients with stage D HF patients are limited. Also, the accuracy of commonly-used predictive equations for REE, the determinants of REE, and its association with HF severity, remain unclear because of limited reports (6–9). This may cause unrecognized energy intake insufficiency, particularly in patients with advanced HF, leading to the development of cachexia.

Serum albumin is often used as a marker reflecting nutritional status or included in indices, such as the Controlling Nutritional Status score, Prognostic Nutritional Index, and Geriatric Nutritional Risk Index (10). However, serum albumin levels indicate the presence of inflammation rather than nutritional status (11). Since patients with HF in a hemodynamically stable condition tend to be dehydrated, serum albumin levels are often maintained until severe malnutrition (12, 13). Consequently, it is often difficult to identify insufficient energy intake by serum albumin levels and more specific biomarkers are needed for this purpose.

The objective of this study was to evaluate the validity of predicted REE values obtained by commonly used formulas in clinical practice and to clarify the determinants of REE in advanced HF. Furthermore, we explored plasma and urinary laboratory markers indicating insufficient energy intake instead of serum albumin levels using measured REE values.

## Methods

### Patients

We planned to measure REE by indirect calorimetry if a right heart catheterization was scheduled in a hemodynamically stable condition in 293 patients who were hospitalized for close investigation or treatment for advanced HF, which was considered by the attending physician according to the HF guidelines (14), at Osaka University Hospital from June 2015 to November 2020. As a result, REE data were successfully obtained in 72 patients. Serum and urinary laboratory samples were collected from 17 of the 72 patients, excluding patients with proteinuria. To analyze the relationship between REE and BMI, we classified the patients into three groups according to BMI (kg/m^2^) as follows: <18.5 in the underweight group, ≥18.5 and <25 in the normal weight group, and ≥25 in the overweight group (15).

This study was approved by the Institutional Review Board (IRB) of Osaka University (IRB number: 16209–2). Informed consent was obtained in the form of an opt-out option on the website.

### Measurement and prediction of REE and TEE

The oxygen consumption (VO_2_) and carbon dioxide production (VCO_2_) were measured using a face-covering mask connected to an indirect calorimetry (AE-100i; Minato Medical Science Co., Osaka, Japan). During the measurement, the patients were in a lying position at rest for at least 20 min after a 4-h fast in a hemodynamically stable phase, defined as no requirement for up-titration of intravenous inotropes or mechanical circulatory supports. REE was calculated from the average of VO_2_ and VCO_2_ during the last 5 min of indirect calorimetry measurements, using the abbreviated Weir equation (16). Other indices related to energy expenditure, including the TEE, were calculated according to previous reports (17–20) (Table 1). REE per body weight (REEBW) was evaluated because it is commonly used to consider the required energy in routine clinical practice. In this study, the activity index was set at 1.3 because the hemodynamically stable inpatients with HF were physically active away from the bed (20). We modified the European Society for Clinical Nutrition and Metabolism (ESPEN) formula by adjusting the coefficients with obtained REEBW data and proposed it as a new predictive formula for TEE, indicating the energy required per day (Table 1).

**Table 1:** Abbreviations and their calculation.

Abbreviation	Definition	Calculation
REE, kcal/day	Resting energy expenditure using the Weir equation (16)	5.616 × VO_2_ + 1.584 × VCO_2_
pREE (kcal/day)	Predicted REE using the Harris–Benedict equation (17)	Male pREE = 66.5 + 13.75 × W + 5.003 × H – 6.775 × Ag
		Female pREE = 655.1 + 9.563 × W + 1.850 × H – 4.676 × Ag
REEBW (kcal/kg/day)	REE per body weight	REE/body weight
pREEBW (kcal/kg/day)	Predicted REE using the Harris–Benedict equation per body weight (17)	pREE/body weight
TEE (kcal/day)	Total energy expenditure	REE × 1.3 (activity index)
pTEE (HB) (kcal/day)	Predicted TEE using the Harris–Benedict equation (17)	pREE×1.3 (activity index)
pTEE (ESPEN) (kcal/day)	Predicted TEE using the ESPEN formula (18)	25 × body weight
pTEE (Mifflin-St Joer) (kcal/day)	Predicted TEE using the Mifflin-St Joer equation (19)	Male pTEE = 1.3 × (10 × W + 6.25 × H – 5 × Ag + 5)
		Female pTEE = 1.3 × (10 × W + 6.25 × H – 5 × Ag – 161)
pTEE (proposed formula) (kcal/day)	Predicted TEE using body weight and coefficients calculated in each BMI classification (modified ESPEN formula)	mean REEBW obtained in each BMI classification × 1.3 (activity index) × body weight

### Right heart catheterization

We performed right heart catheterization based on clinical indication in a hemodynamically stable condition to evaluate cardiac function implying pathophysiological information. Hemodynamic parameters, right atrial pressure (RAP), which mainly reflects right heart function (21), pulmonary arterial wedge pressure (PAWP), which mainly reflects left heart function, and venous oxygen saturation (SvO2) and cardiac index by Fick’s formula (22), which reflect both heart functions, were measured.

### Sample collection

Blood samples were collected before breakfast after fasting for at least 8 h, and centrifuged to obtain serum or plasma separately. Urine samples were collected from urine stored for 24 h overnight before blood sample collection. Plasma and urinary amino acid (AA) analyses were performed using liquid chromatography/mass spectrometry and high-performance liquid chromatography (23).

### Evaluation of food intake

The amounts of macronutrients were determined for each type of hospital meal. Energy and protein intakes were calculated from the records of the type and intake of each meal recorded by the nurses in the electronic medical record. The average of the consecutive 3 days prior to the day of blood sample collection was used for the analysis (24).

### Statistical analyses

Statistical analyses were performed using International Business Machines Statistical Package for the Social Sciences Statistics (version 26). The model assumption of normality was assessed and confirmed using the Shapiro–Wilk test and Q–Q plots of residuals. Continuous variables are expressed as mean ± standard deviation or median (interquartile range), as appropriate. The paired or unpaired t test was used for comparisons between the two groups. The 2-way ANOVA without replication was used to investigate the effects of BMI category and sex on REEBW and the differences between TEE and pTEE by each equation.

Correlations between two variables were analyzed by Pearson’s or Spearman’s rank correlation coefficient test, as appropriate, and each correlation coefficient is expressed as *r*. Multiple regression analysis was performed using the forced-entry model to estimate associations between REE and clinical parameters related to HF severity, with adjustment for body weight.

## Results

### The determinants and comparison of REE with the value by the predictive equation

**Figure 1:**
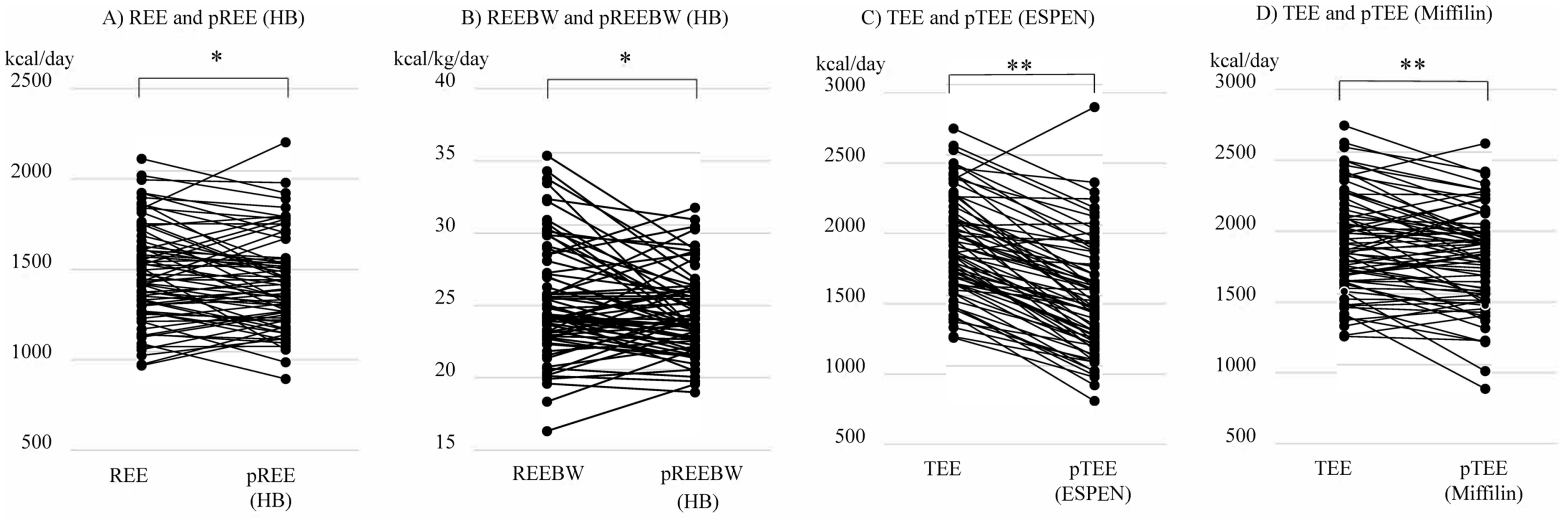
Comparison among **(A)** resting energy expenditure (REE) with predicted value of REE (pREE) by the Harris-Benedict (HB) equation, **(B)** REE per body weight (REEBW) with predicted value of REE per body weight (pREEBW) by the Harris-Benedict (HB) equation, **(C)** total energy expenditure (TEE) and predicted value of TEE (pTEE) by the European Society for Clinical Nutrition and Metabolism (ESPEN) formula, and **(D)** TEE and predicted value of TEE (pTEE) by the Mifflin-St Jeor equation. *p < 0.05, **p < 0.01 by paired *t*-test.

Patient characteristics are summarized in Table 2. This study primarily included patients with advanced HF, as evident in 43 (60%) patients on the transplant waiting list and 61 (85%) patients with stage D HF. REE and REEBW were significantly higher than the predicted values of REE (pREE) and REEBW (pREEBW) by the Harris-Benedict (HB) equation (*p* < 0.05 and *p* < 0.05, respectively, Figures 1A,B). TEE was also significantly higher than the predicted values of TEE (pTEE) based on the ESPEN formula (*p* < 0.01, Figure 1C) or the Mifflin-St Jeor equation (*p* < 0.01, Figure 1D). In the analysis of each sex, the difference between REE/REEBW and pREE/REEBW by HB equation was significant in men (*p* < 0.05, *p* < 0.01, respectively), but not in women (*p* = 0.457, *p* = 0.488, respectively). In contrast, the difference between TEE and pTEE using the ESPEN or Mifflin-St Jeor equations remained significant in both sexes (all *p* < 0.05). The average individual differences between REE and pREE by the HB equation was 49.0 ± 169.4 kcal/day (male: 59.0 ± 177.3 kcal/day, female: 24.8 ± 149.6 kcal/day). Such individual differences were also observed between REEBW and pREEBW by the HB equation (1.0 ± 0.4 kcal/kg/day) or between TEE and pTEE by the ESPEN formula (373.9 ± 243.5 kcal/day) or Mifflin-St Jeor equations (106.7 ± 216.3 kcal/day).

**Table 2:** Clinical characteristics of patients with chronic heart failure.

	Total	Male	Female
Number, *n*	72	51	21
Demographics			
Age, years	50 ± 13	51 ± 12	47 ± 15
Anthropometrics			
Body Mass Index, kg/m^2^	19.9 (6.2)	21.3 (4.5)	20.1 (4.1)
Grip strength, kg	28.2 ± 8.90	31.1 ± 7.96**	21.3 ± 7.16**
Knee-extension strength, Nm/kg	31.0 ± 11.04	33.0 ± 9.83 *	25.8 ± 12.44*
Functional class, *n*			
NYHA functional class, II/III/IV	13/34/25	9/25/17	4/9/8
The ACC/AHA stages of heart failure, C/D	11/61	9/42	2/19
Etiology, *n*(%)			
Dilated cardiomyopathy	35 (49)	28 (55)	7 (33)
Ischemic cardiomyopathy	9 (12)	8 (16)	1 (5)
Hypertrophic cardiomyopathy	9 (12)	3 (6)	6 (29)
Others	19 (26)	12 (24)	7 (33)
Medications			
Intravenous inotropic support, *n* (%)	42 (58)	30 (59)	12 (57)
Blood			
AST, IU/l	22 (11)	24 (12)	26 (8)
g-GTP, IU/l	100 (148)	106 (134)	86 (180)
Creatinine, mg/dl	1.09 (0.49)	1.19 (0.54)	0.84 (0.23)
Lymphocytes, /ml	1,406 (549)	1,343 (522)	1,560 (595)
Total cholesterol, mg/dl	168 ± 42.5	162 ± 44.9	180 ± 34.9
Albumin, g/dl	3.9 (0.5)	3.8 (0.5)**	4.1 (0.2)**
CRP, mg/dl	0.38 (0.71)	0.45 (0.82)*	0.19 (0.25)*
BNP, pg/ml	324.1 (411.65)	398.0 (314.88)	324.6 (195.12)
Hemodynamics and cardiac function			
LV ejection fraction, %	26 (13)	24 (12)	30 (14)
RAP, mmHg	7 (4)	7 (4)	6 (3)
PAWP, mmHg	17 ± 7.3	17 ± 7.6	18 ± 6.2
CI, L/min/m^2^	2.3 (0.68)	2.4 (0.70)*	2.1 (0.54)*
Others			
On heart transplantation waiting list, *n*(%)	43 (60)	28 (55)	15 (71)

Categorical variables are represented as number (%). Continuous variables are represented as mean ± standard deviation or median (interquartile range), as appropriate.

NYHA, New York Heart Association; ACC/AHA, American College of Cardiology/American Heart Association; LV, left ventricular; RAP, right atrial pressure; PCWP, pulmonary artery wedge pressure; CI, cardiac index.

*p < 0.05, **p < 0.01, by unpaired *t*-test.

**Figure 2:**
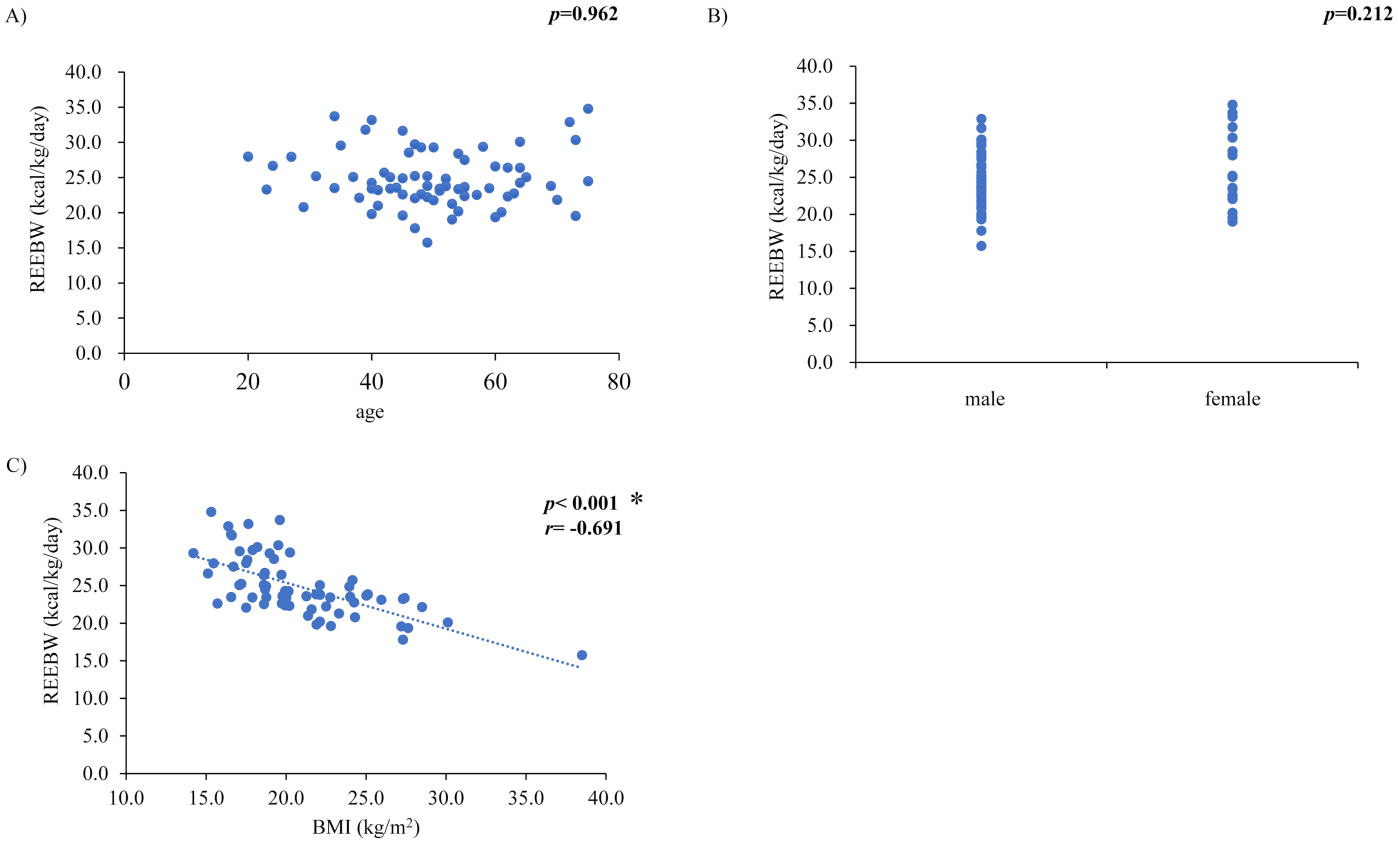
Relationships between resting energy expenditure per body weight (REEBW) and **(A)** age, **(B)** sex, and **(C)** body mass index (BMI) *p < 0.01, *r*: Pearson’s or Spearman’s coefficient value, *p*-value in **(B)** by unpaired *t*-test.

**Figure 3:**
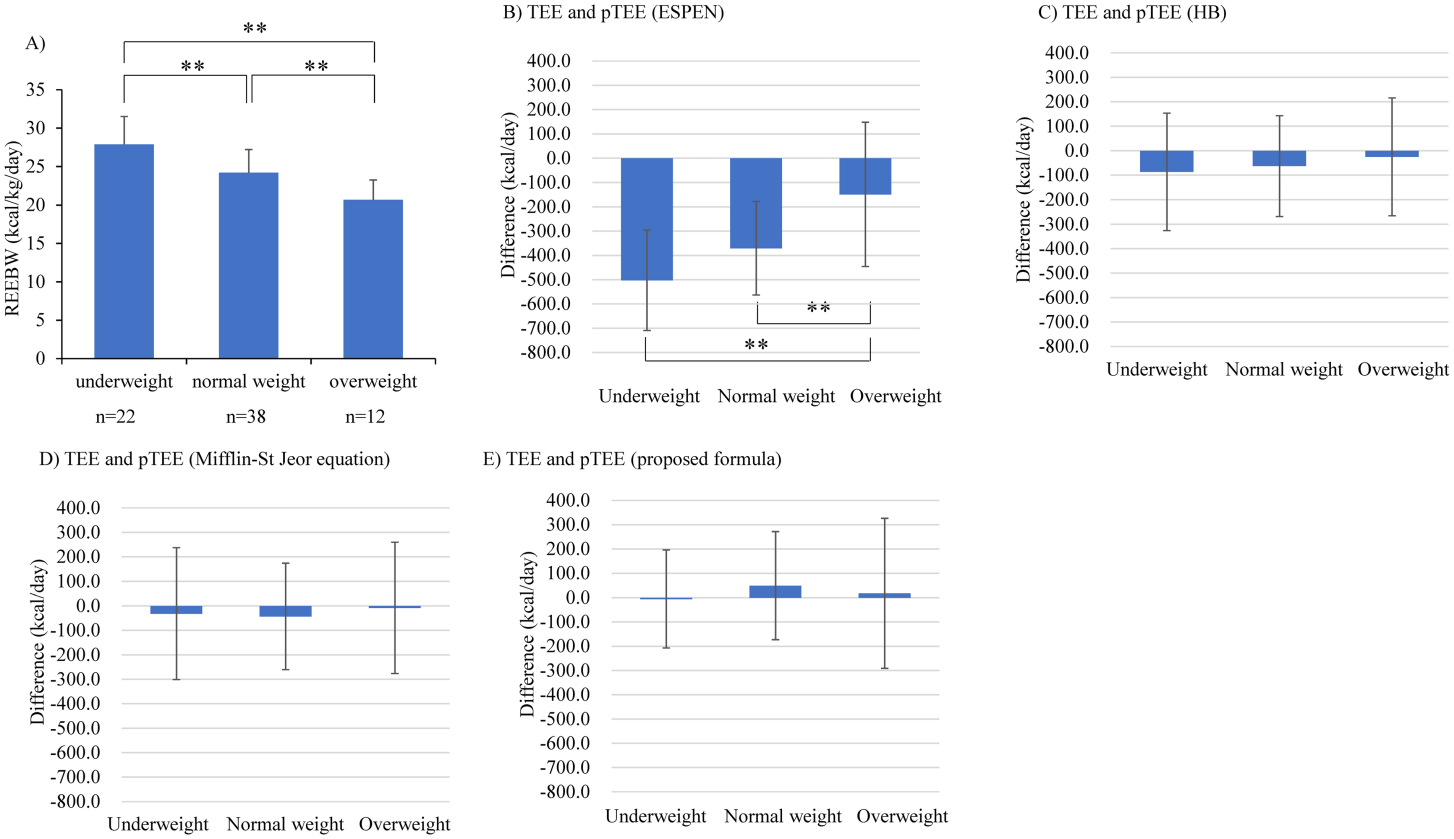
Comparison of **(A)** resting energy expenditure per body weight (REEBW), **(B)** the difference between TEE and pTEE by the European Society for Clinical Nutrition and Metabolism (ESPEN) formula, **(C)** the difference between total energy expenditure (TEE) and predicted value of TEE (pTEE) by the Harris-Benedict (HB) equation, **(D)** the difference between TEE and pTEE by the Mifflin-St Jeor equation, and **(E)** the difference between TEE and pTEE (proposed formula), among each group divided by body mass index. The error bar represents SD **p < 0.01 by 2-way ANOVA test.

The REEBW was not associated with clinical parameters related to hemodynamics and HF severity or inotropes use (mean blood pressure, *p* = 0.818; heart rate, *p* = 0.376; pulmonary artery wedge pressure, *p* = 0.400; right atrial pressure, *p* = 0.843; cardiac index, *p* = 0.599; mixed SvO_2_, *p* = 0.752; log brain natriuretic peptide [BNP], *p* = 0.352; inotropes use, *p* = 0.758). These associations were confirmed by analyses using multiple regression models, where REE was not associated with the above parameters after adjusting for BW. REEBW was not associated with age and sex (Figures 2A,B) but was significantly associated with BMI (*r* = −0.691, *p* < 0.001, Figure 2C). The significant association of BMI with REEBW remained after adjusting for sex. Comparisons of REEBW among each group classified by BMI showed a higher REEBW in the underweight group than in the normal- and overweight groups and a higher REEBW in the normal-weight group than in the overweight group (Figure 3A). The mean REEBW was approximately 28 kcal/kg/day, 24 kcal/kg/day, and 21 kcal/kg/day for underweight, normal-, and overweight groups, respectively, and the coefficients for pTEE (proposed formula) in each BMI group were 36 kcal/kg/day for underweight patients, 32 kcal/kg/day for normal weight patients, and 27 kcal/kg/day for overweight patients. The difference between TEE and pTEE by the ESPEN formula was large and was the greatest in the underweight group (502.4 ± 206.9 kcal/day) and greater in the normal-weight group (370.6 ± 192.5 kcal/day) than in the overweight group (148.8 ± 296.8 kcal/day) (Figure 3B). In contrast, there was no significant difference between TEE and pTEE by the HB equation, the Mifflin-St Jeor equation, and the proposed formula among each BMI group (Figures 3C–E). These associations remained after adjusting for sex.

### Biomarkers for evaluating the energy balance between expenditure and intake in patients with heart failure

**Figure 4:**
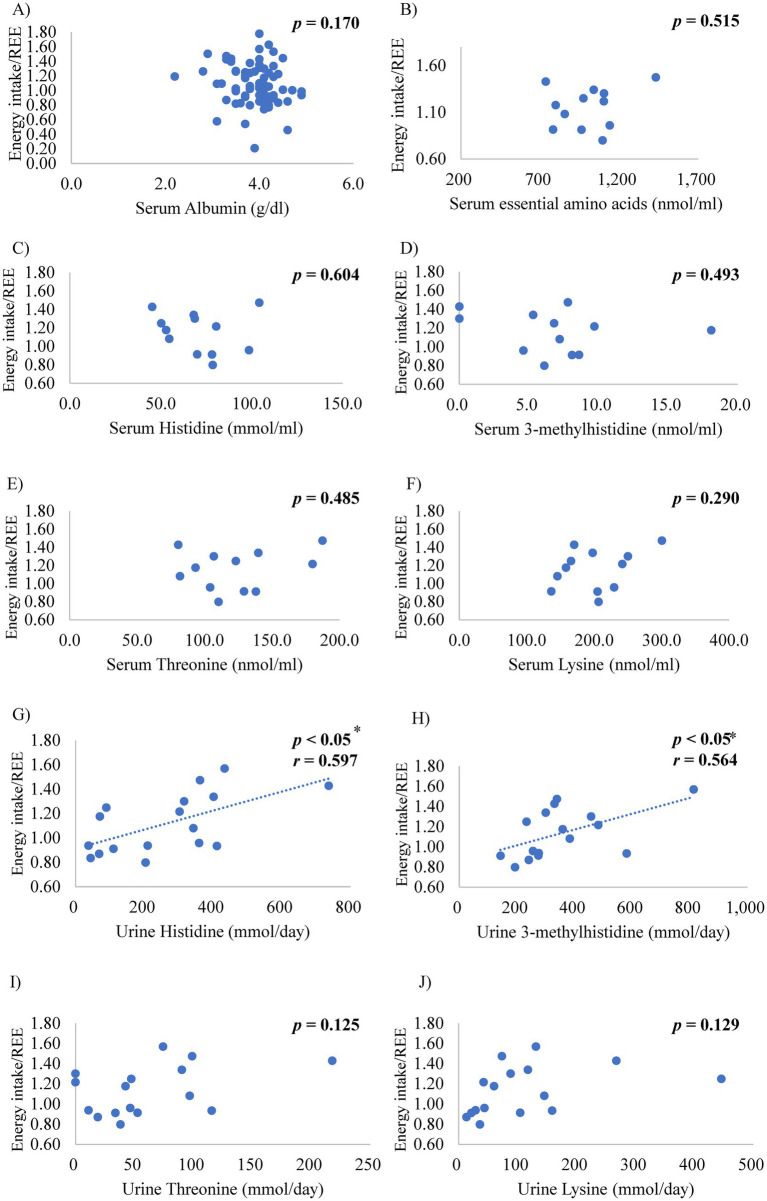
Relationships between the ratio of energy intake to resting energy expenditure (REE) and the serum levels of **(A)** albumin, **(B)** essential amino acids, **(C)** histidine, **(D)** 3-methylhistidine, **(E)** threonine, and **(F)** lysine, and urinary levels of **(G)** histidine, **(H)** 3-methylhistidine, **(I)** threonine, and **(J)** lysine *p < 0.05, *r*: coefficient value by Pearson’s correlation analysis, *p*-value by Pearson’s correlation test.

Table 3 shows the relationships between the candidate biomarkers and the ratio of daily energy intake to REE, protein intake, representative variables of HF features, and renal function. The serum albumin and plasma AA levels, including fractional concentrations, such as essential AA, branched-chain AA, and each essential AA concentration, were maintained in the normal range and had no significant correlation with the ratio of daily energy intake to REE (Figures 4A–F). In contrast, the urinary essential AA excretion was mostly below the normal range. Notably, urinary excretions of histidine and 3-methylhistidine were significantly correlated with the ratio of daily energy intake to REE (Figures 4G,H). Urinary threonine and lysine excretion tended to be positively correlated with the ratio of daily energy intake to REE (Figures 4I,J). These significant correlations remained after adjusting for sex.

**Table 3:** Correlations among albumin, plasma amino acids or urinary amino acids, and associated factors.

	Value	Normal range	Energy intake/REE		Protein intake (g/kg/day)		Log BNP		CI (L/min/m^2^)		eGFRcys (mL/min)	
			*r*	*p*-value	*r*	*p*-value	*r*	*p*-value	*r*	*p*-value	*r*	*p*-value
**Serum**	g/dL	g/dL										
Albumin	4.0 ± 0.40	3.8–5.2		0.308		0.633		0.655	−0.298	0.015^*^		0.426
**Plasma**	nmol/mL	nmol/mL										
Total amino acids	2909.7 ± 425.69	2068.2–3510.3		0.623		0.548		0.401		0.79		0.333
Essential amino acids	984.2 ± 196.75	660.0–1222.3		0.323		0.621		0.261		0.502		0.168
Branched chain amino acids	434.2 ± 86.46	265.8–579.1		0.304		0.373		0.268		0.832		0.059
Histidine	69.5 ± 18.41	59.0–92.0		0.894		0.41		0.669		0.718		0.276
Phenylalanine	69.3 ± 18.21	42.6–75.7		0.52		0.997	0.694	0.008^**^		0.226		0.288
Tryptophan	63.0 ± 14.50	37.0–74.9		0.951		0.634	0.73	0.005^**^		0.157		0.367
Threonine	121.0 ± 33.77	66.5–188.9		0.345		0.481		0.621		0.781		0.752
Lysine	197.3 ± 47.95	108.7–242.2		0.198		0.111		0.3		0.556		0.244
Valine	226.5 ± 43.69	147.8–307.0		0.309		0.626		0.336		0.751		0.078
**Urine**	mmol/day	mmol/day										
Histidine	265.0 ± 186.70	436.4–2786.5	0.556	0.021^*^	0.556	0.025^*^		0.107		0.771		0.257
Phenylalanine	20.0 ± 21.71	27.2–110.2		0.184		0.83		0.152		0.13		0.715
Tryptophan	19.4 ± 28.05	20.7–150.7		0.13		0.549		0.292		0.233		0.827
Threonine	59.4 ± 53.83	79.9–528.3		0.088		0.485		0.156		0.427		0.923
Lysine	105.9 ± 109.30	51.6–1639.6		0.086		0.886		0.766		0.538		0.784
Leucine	17.1 ± 16.35	24.6–89.3		0.711		0.852		0.11	0.675	0.004^**^		0.803
3-methylhistidine	345.7 ± 164.11	113.4–480.9	0.539	0.031^*^	0.537	0.026^*^		0.909		0.403		0.729

Units of each parameter are as follows: g/dL for serum albumin, nmol/mL for serum amino acids, and mmol/day for urinary amino acids.

*r*: Pearson correlation coefficient, *p-*value by Pearson’s correlation test, ^*^p < 0.05, ^**^p < 0.01.

REE, resting energy expenditure; CI; cardiac index; eGFRcys, cystatin C-based glomerular filtration rate.

## Discussion

This study has three major findings. First, REE, REEBW, and TEE by indirect calorimetry in hospitalized patients with advanced HF were significantly higher than the values predicted by the HB male equation, ESPEN formula, or Mifflin-St Jeor equation, which are well-used clinical equations. Second, REEBW was inversely correlated with BMI but not with clinical parameters associated with HF severity or hemodynamics. The pTEE by the ESPEN formula was the most underestimated in the underweight group. The mean REEBW for all patients was 25 kcal/kg/day, while for the underweight (<18.5 kg/m^2^) it was 28 kcal/kg/day. This finding suggests that underweight patients with HF require more energy than predicted by commonly-used predictive equations. The energy requirements per kg that were calculated with activity index and REEBW in each BMI group were 36 kcal/kg/day for the underweight group, 32 kcal/kg/day for the normal weight group, and 27 kcal/kg/day for the overweight group. These coefficients by BMI category, not a constant coefficient, may be useful in daily clinical practice. Finally, the excreted amount of urinary essential AAs was mostly below the normal range, whereas serum albumin and plasma essential AA levels were within the normal range. Urinary levels of histidine and 3-methylhistidine correlated with the ratio of caloric intake to REE, indicating that urinary essential AAs may become a possible biomarker for energy balance.

The REE is affected by age, sex, body composition, and organ metabolism (25, 26). Previous studies have shown higher REE and lower fat-free mass in patients with HF than in healthy controls (27). At rest, the brain, liver, heart, and kidneys account for approximately 60–70% of REE in adults, specifically the heart for approximately 10%, whereas skeletal muscle accounts for 20–30% of REE (25, 28). Factors that increase cardiac oxygen consumption, such as heart rate, afterload, cardiac contractility, and inotropic use, can increase cardiac energy expenditure (29). We could not determine the associations between the factors increasing cardiac oxygen consumption and REE, at least in a hemodynamically stable condition. The negative correlation between BMI and REEBW can be partially explained by the fact that the energy expended by organs metabolizing at rest, such as the heart, brain, liver, and kidney, is relatively high (25) in patients with low body weight. According to expert opinion in the guidelines, isocaloric nutrition rather than hypocaloric nutrition is recommended after the early phase of acute illness (18), and the use of a published predictive equation or a simplistic weight-based equation (25–30 kcal/kg/day) is recommended to determine energy requirements in the absence of indirect calorimetry (30). In the previous study (6), the mean REEBW was 22 kcal/kg in patients with normal nutritional status and 24 kcal/kg in those with low nutritional status. In contrast, the mean REEBW was approximately 25 kcal/kg in our cohort, which consisted mainly of stage D HF patients, and 28 kcal/kg in the underweight patients. This finding suggests the risk of underestimating energy requirements, especially in underweight patients with advanced HF. Recognizing the association between energy requirements and BMI would be helpful for nutritional management, especially in patients with advanced HF and on the heart transplantation waiting list.

Our study showed that urinary histidine and 3-methylhistidine excretion decreased in proportion to decreased energy intake, whereas plasma histidine levels were maintained. Urinary 3-methylhistidine excretion is used as a marker of muscle catabolism because excretion occurs without recycling once 3-methylhistidine-containing myofibrillar proteins, such as actin and myosin, are degraded (31). Our results suggest that the increased demand for histidine is caused by mechanisms other than the consumption of histidine by muscle catabolism. Histidine is known to have several roles in proton buffering, scavenging of reactive oxygen and nitrogen species, erythropoiesis, metal ion chelation, and the histaminergic system (31). It is distributed as histidine-containing dipeptides (e.g., carnosine), histidine-rich proteins (e.g., hemoglobin), and histidine metabolites (e.g., 3-methylhistidine). Carnosine is composed of histidine and *β*-alanine, which is abundant in the skeletal muscle (31). The carnosine level is higher in fast-twitch muscle fibers than in slow-twitch muscle fibers, suggesting that carnosine plays a proton-buffering role against reduced pH due to the lactic acid accumulation that occurs in fast-twitch muscle fibers during exercise under anaerobic conditions (31). The fast-twitch muscle fibers increase with the decrease in slow-twitch muscle fibers that occurs in HF (32). One hypothesis based on our findings is that the urinary histidine decrease may indicate an increased demand for carnosine which is synthesized from histidine and β-alanine. Another unique pathophysiology of HF, such as increased oxidative stress and anemia, may be responsible for the increased demand for histidine. The consumption of other essential AAs would be increased in patients with HF because their excretion in the urine decreases below the normal range. In turn, supplementation with these AAs, especially histidine, would be an effective nutritional strategy for patients with HF (33–37). In our study, phenylalanine and tryptophan levels in the plasma and urinary leucine excretion were associated with log BNP and cardiac index, respectively. However, no associations with nutritional intake were observed. Previous studies have shown a correlation between specific AA concentrations and cardiac function that reflects pathological metabolic changes in patients with HF (38–41). The detailed mechanism of the specific AA profile in HF is yet to be elucidated; however, AA profiles are modified by multiple factors. Our study is the first to show that the urinary AA pattern observed in patients with HF is associated with nutritional intake. The decreased urinary excretion of essential AAs, specifically histidine, would be a useful biomarker to detect insufficient energy intake prior to a decrease in plasma albumin or AA level.

### Limitations

We performed REE measurements 4 h after meals because of practical reasons. For REE measurements, a minimum fast of 5 h after meals and 4 h after a small meal is recommended if further fasting is clinically inappropriate. The peak of the increase in metabolic rate associated with the diet occurs between 60 and 180 min. Therefore, there may be a diet-induced metabolic rate increase in measured REE. However, measurement 4 h after small meals is considered acceptable (42). REE is generally reported to be negatively correlated with age, decreasing in individuals aged over 60 years (26). Our observation that age was not associated with REE may be attributed to the relatively young age of our study population, with an average age of 50 years, because heart transplantation candidates accounted for 60% of the patients (Table 2). Furthermore, in our advanced HF cohort, there were few patients (5 of 72) with HF and preserved ejection fraction, which is common in older patients with HF. The single-center design and small study sample limited the statistical power, resulting in possible type I and II errors. The mechanism of AA profile change and its effectiveness for nutritional management need further investigation.

## Conclusion

Patients with advanced HF, especially those with a low BMI, require more energy per weight due to elevated REE. Inadequate nutritional intake can be identified by decreased urinary excretion of essential AAs, especially histidine, even when serum albumin and plasma AA concentrations do not decline.

## Data Availability

The original contributions presented in the study are included in the article/supplementary material, further inquiries can be directed to the corresponding author.
